# Germination Capacity of Five Dry-Forest Tree Species from Southern Ecuador After 12 to 17 Years of Ambient Laboratory Storage

**DOI:** 10.3390/plants15213381

**Published:** 2026-11-01

**Authors:** Jasmine Cañar, Diego P. Vélez-Mora, Elizabeth Gusmán-Montalván

**Affiliations:** 1 Carrera de Biología, Universidad Técnica Particular de Loja, Loja 1101608, Ecuador; 2 Departamento de Ciencias Biológicas y Agropecuarias, Universidad Técnica Particular de Loja, Loja 1101608, Ecuador; ecguzman@utpl.edu.ec

**Keywords:** seed longevity, seed aging, ex situ conservation, germination speed, desiccation tolerance, Tumbesian region, ambient seed storage, ecological restoration

## Abstract

Ex situ seed collections support dry-forest restoration, yet how much germination capacity tropical tree seeds retain under ambient, non-refrigerated storage is rarely documented beyond a few years. We compared historical seed lots of five native tree species from the Tumbesian dry forests of southern Ecuador, kept for 12 to 17 years in paper bags under uncontrolled laboratory conditions, with freshly collected lots, sowing 948 seeds. Because the lots are independent collections without initial germination data, all comparisons are observational and describe retained germination capacity rather than isolated storage effects; ungerminated seeds were not tested for viability. Historical lots germinated less than fresh lots in four species (58–87% lower); overall, 15.6% of historical and 48.1% of fresh seeds germinated. The difference was largest in *Ceiba trichistandra* (10.4% vs. 81.2%) and absent in *Caesalpinia spinosa* (25.0% vs. 27.1%), whose low fresh-lot germination may reflect physical dormancy. *Coccoloba ruiziana*, regionally listed as recalcitrant, still germinated at 23% after 14 years, which is inconsistent with recalcitrant behavior and supports treating it as intermediate. Relative loss increased with years in storage, but this exploratory association is confounded with species and collection year. Only *C. ruiziana* produced clearly shorter seedlings from historical seed. Decade-old ambient holdings can still yield seedlings, but sowing quantities must be species-specific.

## 1. Introduction

Tropical dry forests (TDF) are the most threatened tropical forest biome [1,2], and in the Tumbesian dry forests of southern Ecuador and northwestern Peru, an average of 46% of each species’ range across the 50 commonest trees is already highly vulnerable to fire, habitat conversion, overgrazing, overexploitation or climate change [3]. In Ecuador, the TDFs of the southern province of Loja support high plant diversity and endemism and provide critical ecosystem services to local communities [4,5]. Their natural regeneration, however, is constrained by chronic grazing pressure and by the short establishment window imposed by strong rainfall seasonality [6,7]. Restoring them therefore depends on a reliable supply of propagules, which in these seasonal systems rests in practice on ex situ seed collections capable of storing germplasm across years to decades [3,8]. The value of such collections, however, hinges critically on whether historical seeds retain germination capacity over storage horizons relevant to restoration planning, a question that, for most tropical species, remains empirically untested beyond a few years of storage.

Seeds age during prolonged storage because reactive oxygen species (ROS) accumulate even in the metabolically quiescent dry state. The resulting oxidative damage to lipids, proteins and nucleic acids progressively impairs reserve mobilization and radicle cell elongation [9,10,11,12]. Deterioration then follows a graded sequence. Early damage reduces germination speed and synchrony while overall viability is still maintained, and only in later stages does viability itself collapse [13,14]. The trajectory and rate of aging depend on seed composition, moisture content and storage temperature and vary substantially among species and even among populations of the same species [13,15,16]. Seeds classified as orthodox (tolerant of desiccation to low moisture contents) are generally assumed storable for years to decades, whereas intermediate seeds (desiccation-sensitive) are expected to lose viability rapidly under conventional dry storage [17]. This dichotomy, however, captures only the endpoint of storability and says little about how quickly vigor deteriorates within the “storable” category itself.

Germination percentage (GP) measures the germination capacity of a seed lot under the test conditions; it equals viability only when every viable seed is non-dormant, and the test supplies the conditions it requires [10]. Mean germination time (MGT) and the median germination time of germinants (T50g) describe the timing of germination among the seeds that germinate; when final germination differs strongly between lots, they summarize different fractions of each lot and are not independent measures of lot vigor [18]. The germination speed index (GSI) combines both dimensions, because it increases with germination speed and with the number of germinants [19]. Both dimensions matter for restoration. Low GP reduces the effective seed supply available per sowing event, while slower and less synchronous germination lengthens nursery production cycles and may compromise seedling establishment during the narrow soil-moisture windows characteristic of TDF rainfall seasonality [8]. Seed morphometric traits, and seed weight in particular, influence reserve allocation to the embryo and can therefore modulate both germination speed and early seedling growth [20,21].

Despite this pressing restoration need, the effects of long-term storage (more than five years) on the germination capacity of native TDF species in Ecuador have never been evaluated. Silveira et al. [22] documented maintained viability but altered germination timing in *Mimosa foliolosa* after 36 months of storage. Beyond that window, what is known about ambient, non-refrigerated storage comes almost entirely from crop species and from orthodox seeds of temperate origin [15,16]. Whether these patterns extend to Neotropical TDF trees held at ambient conditions for 12 to 17 years, the timescales already accumulated in several institutional seed collections in the region, remains unknown. Here we ask how much germination capacity remains in historical seed lots of five dry-forest tree species from Loja Province after 12 to 17 years of ambient laboratory storage, relative to contemporary lots of the same species collected with the same protocol. Because historical and contemporary lots are independent collections, this comparison describes retained germination capacity; it cannot estimate how much storage itself reduced germination. We measured germination percentage, germination timing and seed morphometry and followed the resulting seedlings to ask whether differences between lots extend beyond germination into early growth. We examined three expectations. First, historical lots would show lower germination percentage and slower germination than contemporary lots in most species. Second, if *Coccoloba ruiziana* were intermediate, as listed in a regional compilation [23], its historical lot would retain little or no capacity to germinate after more than a decade of dry storage, given the desiccation sensitivity of intermediate seeds [17]; with a single such species, this expectation can only be examined qualitatively. Third, if larger seeds were buffered against deterioration, the relationship between seed mass and germination would differ between historical and contemporary lots.

## 2. Results

### 2.1. Seed-Lot Characterization

We first describe how the historical and fresh lots differed in seed size, since any difference in germination must be read against the material each lot provided (the five species and their seed lots are listed in Table 1). Seed size differed between lots in four of the five species, but not in a consistent direction (Table S1, Figure S1). The largest difference was in *E. ruizii,* whose historical seeds weighed 43% less than the fresh ones (mass ratio 0.57, 95% CI 0.52–0.62) and were also shorter and narrower. *C. spinosa* and *C. trichistandra* showed smaller differences in mass, of about 5% (0.945, 0.914–0.978 and 0.953, 0.922–0.986) but diverged in shape: historical *C. spinosa* seeds were 12% shorter, whereas historical *C. trichistandra* seeds were 8% longer and slightly narrower. *G. spinosa* ran counter to the rest, its historical seeds being 38% heavier (1.38, 1.29–1.48) as well as longer and wider. *C. ruiziana* differed in no trait. Because the historical and fresh lots come from different collection years and, in part, from different mother trees, these contrasts describe the lots as received rather than the effect of storage. The inconsistent direction among species reinforces that reading: uniform desiccation and reserve loss during storage would reduce seed mass in every species, and in *G. spinosa* it increased.

### 2.2. Germination Percentage

**Figure 1:**
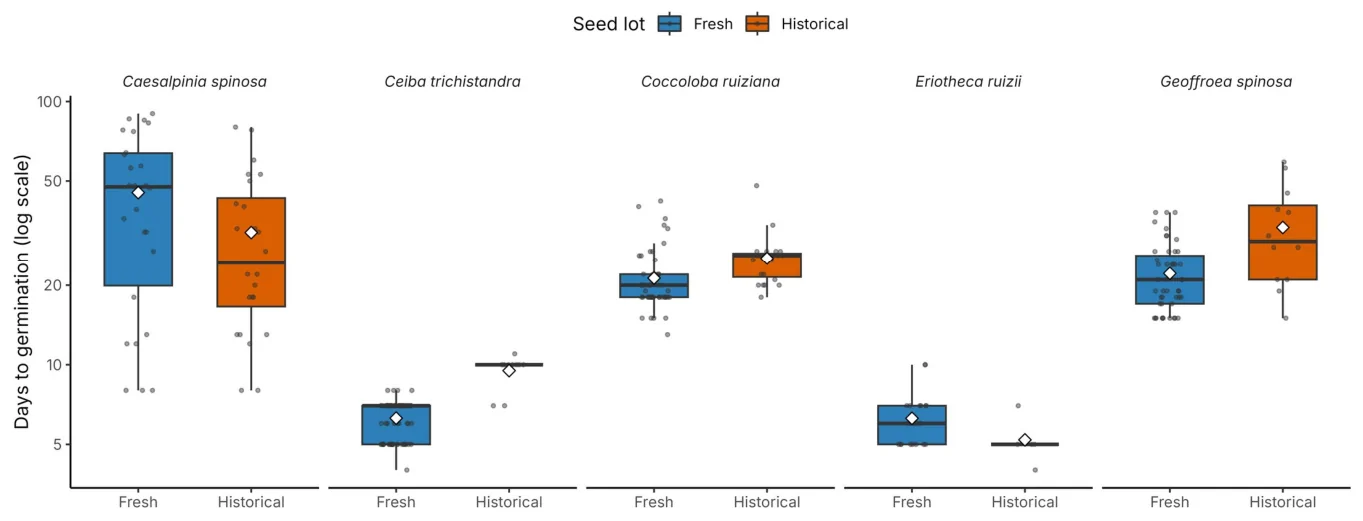
Days to germination of germinated seeds of five dry-forest tree species from historical (orange) and fresh (blue) seed lots. Boxes show the median and interquartile range, whiskers extend to 1.5 times the interquartile range, gray points are individual seeds, and white diamonds mark the mean germination time. The vertical axis is on a common logarithmic scale. Historical lots germinated later in *C. trichistandra*, *C. ruiziana* and *G. spinosa*, and earlier in *C. spinosa* and *E. ruizii*.

Germination was lower in the historical lot than in the fresh lot in four of the five species, by amounts that differed greatly among them (lot, χ^2^ = 127.2, d.f. = 1, *p* < 0.001; species × lot, χ^2^ = 49.5, d.f. = 4, *p* < 0.001). Across all species, 15.6% of historical seeds germinated against 48.1% of fresh seeds (Figure 1). The largest difference was in *C. trichistandra*, from 81.2% to 10.4%, a loss of 70.8 percentage points and of 87.2% of the fresh-lot value (odds ratio 0.027, 95% CI 0.012–0.062). *E. ruizii* went from 22.9% to 5.2% (17.7 points, 77.3% relative loss; OR 0.185, 0.067–0.512), *G. spinosa* from 54.7% to 14.0% (40.7 points, 74.5%; OR 0.135, 0.064–0.283) and *C. ruiziana* from 55.0% to 23.0% (32.0 points, 58.2%; OR 0.244, 0.133–0.450). *C. spinosa* was the exception: it germinated at 25.0% in the historical lot and 27.1% in the fresh lot, and its odds ratio of 0.90 has a confidence interval spanning one (0.47–1.71). Low germination in both lots of this hard-seeded species may reflect physical dormancy (Section 3.1).

### 2.3. Desiccation-Tolerance Class and Years in Storage (Exploratory)

The historical lot of *C. ruiziana*, the only species listed as recalcitrant, retained 23.0% germination after 14 years of dry storage and ranked fourth of five in relative difference from its fresh lot, behind three species classified as orthodox. With a single putatively recalcitrant species, this ranking is an observational comparison, not a test of desiccation-tolerance class. Ranked by years in storage, relative differences ranged from 7.7% in *C. spinosa* (12 years) to 87.2% in *C. trichistandra* (17 years) (Spearman ρ = 0.975; Figure S3). This association is descriptive only. Each point in Figure S3 represents, at the same time, a different species, collection year, storage duration and set of mother trees, and the two sowing groups are represented unequally; *n* = 5 species are not independent replicates. The figure is therefore not a seed-survival curve and provides no estimate of storage longevity or aging kinetics. It shows only that our data cannot separate desiccation-tolerance class from years in storage, because *C. ruiziana* happened to be among the more recently collected lots.

### 2.4. Germination Timing

**Figure 2:**
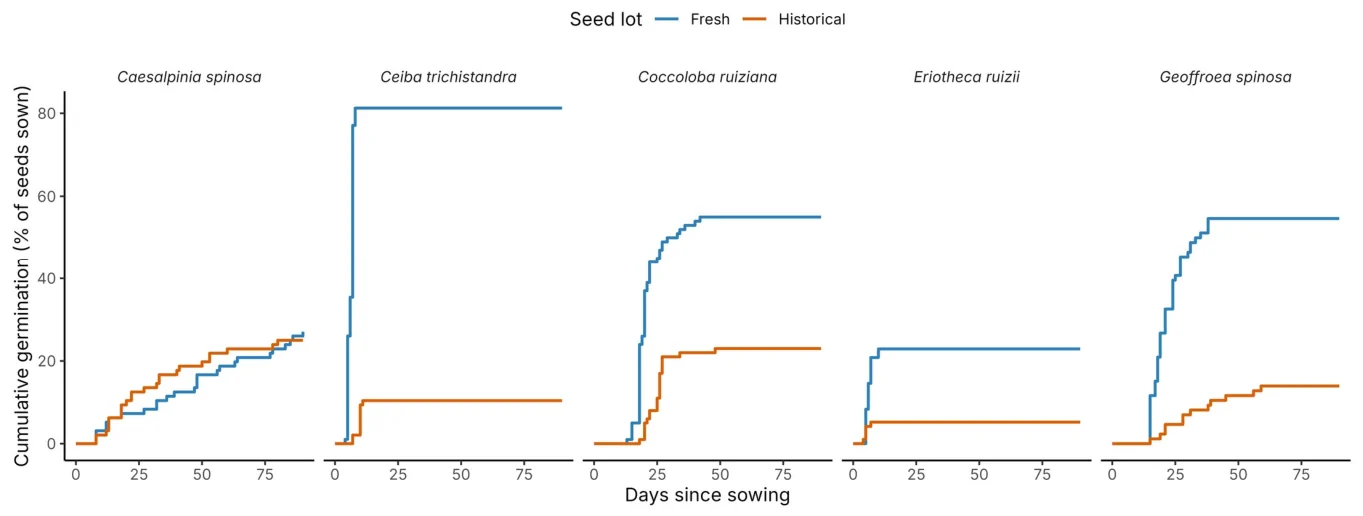
Cumulative germination of five dry-forest tree species from historical (orange) and fresh (blue) seed lots, as a percentage of the seeds sown. The height each curve reaches is the germination percentage, and its slope is the germination rate, so the figure shows both components of Hypothesis 1 at once.

Historical lots germinated more slowly overall (lot, χ^2^ = 48.2, d.f. = 1, *p* < 0.001), but the size and even the direction of the shift depended on the species (species × lot, χ^2^ = 41.7, d.f. = 4, *p* < 0.001; Table 2, Figure 1). Three species germinated later from historical seed: *C. trichistandra* by 3.2 days (6.3 to 9.5 days; time ratio 1.51, 95% CI 1.36–1.68), *G. spinosa* by 11.2 days (22.2 to 33.3; ratio 1.50, 1.23–1.84) and *C. ruiziana* by 4.1 days (21.3 to 25.4; ratio 1.19, 1.06–1.34). These are the three species with the largest absolute declines in germination percentage, so in them germination percentage and germination timing differed in the same direction. The two remaining species moved in the opposite direction, although neither contrast was clear after correction: *C. spinosa* germinated 13.3 days earlier from historical seed (45.2 to 31.9 days; ratio 0.71, 0.49–1.02) and reached T50g 23 days earlier (47.5 to 24.5 days), and *E. ruizii* germinated 1.1 days earlier (6.3 to 5.2; ratio 0.83, 0.68–1.01). Because these timings refer only to germinated seeds, earlier germination in these two lots may reflect the absence of a slow-germinating fraction rather than faster germination (Section 4.4). The germination speed index, which rises with both speed and the number of germinants, was higher in the fresh lot of four species, most markedly in *C. trichistandra* (12.76 versus 1.08) and *E. ruizii* (3.66 versus 0.99), and marginally higher in the historical lot of *C. spinosa* (1.15 versus 1.05). T50g followed the same pattern as mean germination time in every species (Table 2). Plotting germination cumulatively shows both components at once: the height each curve reaches is the germination percentage and its slope is the rate, so the gap between lots opens early in *C. trichistandra* and *E. ruizii,* which germinate within days, and late in *C. spinosa* and *C. ruiziana*, which germinate over weeks (Figure 2).

The time-to-event analysis, which uses all 948 sown seeds and is our primary analysis of germination timing, gave the same interaction (lot, χ^2^ = 116.5, d.f. = 1, *p* < 0.001; species × lot, χ^2^ = 52.9, d.f. = 4, *p* < 0.001). Because it is not conditional on having germinated, the species-dependent difference between lots is not an artifact of comparing lots whose germination fractions differ by up to eightfold. The model does not separate how many seeds germinate from how fast, so the timing contrasts themselves come from the Gamma model. Per-species contrasts from this model (Table S4) show that, once ungerminated seeds are taken into account, historical lots took longer to germinate than fresh lots in four species: *C. trichistandra* (time ratio 5.42, 95% CI 3.66–8.04), *G. spinosa* (3.45, 2.37–5.03), *C. ruiziana* (2.26, 1.64–3.12) and *E. ruizii* (2.17, 1.37–3.42; Holm *p* ≤ 0.002 in all four), but not in *C. spinosa* (0.94, 0.68–1.32; Holm *p* = 0.73). In *E. ruizii*, therefore, the earlier mean germination of the five historical germinants does not reflect faster germination of the lot as a whole.

### 2.5. Seedling Growth and Final Size

Final shoot height, recorded at each individual’s last measurement, differed between lots in a direction that depended on the species (lot, χ^2^ = 5.07, d.f. = 1, *p* = 0.024; species × lot, χ^2^ = 19.0, d.f. = 4, *p* < 0.001; Table S2, Figure S2). *C. ruiziana* was the one species in which historical seed produced clearly shorter seedlings, by 1.31 cm (95% CI 0.60–2.02 cm shorter; Holm *p* = 0.002). *C. trichistandra* went the other way: its historical-lot seedlings were 2.25 cm taller than the fresh-lot ones (0.43–4.08 cm; Holm *p* = 0.064). *E. ruizii* seedlings from historical seed were 2.03 cm shorter, but the contrast was not clear after correction (Holm *p* = 0.104), and neither *C. spinosa* nor *G. spinosa* differed.

Seedling age at the census accounted for a large part of the variation in height (χ^2^ = 68.6, d.f. = 1, *p* < 0.001), as expected given that historical lots germinated later. Adding it as a covariate did not reverse any contrast: the deficit in *C. ruiziana* fell to 0.80 cm and the excess in *C. trichistandra* rose to 3.03 cm, and both became clearer after correction (Holm *p* = 0.018 in each case). The height differences we observed are therefore not an artifact of the historical lots’ later start.

Leaf number showed no clear lot difference in four species. The model indicated 2.10 times more leaves in the historical lot of *E. ruizii* (1.47–3.00), but that contrast rests on five historical-lot seedlings and the exact test does not support it (Mann–Whitney *p* = 0.58), so we do not interpret it.

Individual mean relative growth rate differed between lots in two species, and in both the historical lot grew faster (lot, χ^2^ = 15.9, d.f. = 1, *p* < 0.001; species × lot, χ^2^ = 36.5, d.f. = 4, *p* < 0.001). Historical-lot seedlings of *C. trichistandra* grew 2.6 times faster than fresh-lot ones (0.033 versus 0.013 cm cm^−1^ day^−1^; Holm *p* < 0.001), and those of *E. ruizii* 1.8 times faster (0.021 versus 0.011; Holm *p* = 0.027, exact Mann–Whitney *p* = 0.026). The remaining three species did not differ.

These two differences are not evidence that storage improved seedling vigor. They occur in precisely the two species whose historical lots germinated least, 10.4% in *C. trichistandra* and 5.2% in *E. ruizii*, and they rest on ten and four seedlings, respectively (one of the five historical *E. ruizii* seedlings had a positive height at a single census only, so its relative growth rate could not be computed), against 78 and 22 from the fresh lots. Seedlings from a lot in which only one seed in ten or twenty germinated are the subset whose seeds retained the capacity to germinate, and faster growth among such a subset is what selection of that kind predicts. The stronger the filter, the more selected the seeds that germinate, and the fact that the effect appears in the two most severely filtered lots and in none of the three where germination held up is consistent with selection rather than with a storage benefit. We report these comparisons for completeness and do not interpret them as effects of storage.

### 2.6. Seed Mass and the Probability of Germination

**Figure 3:**
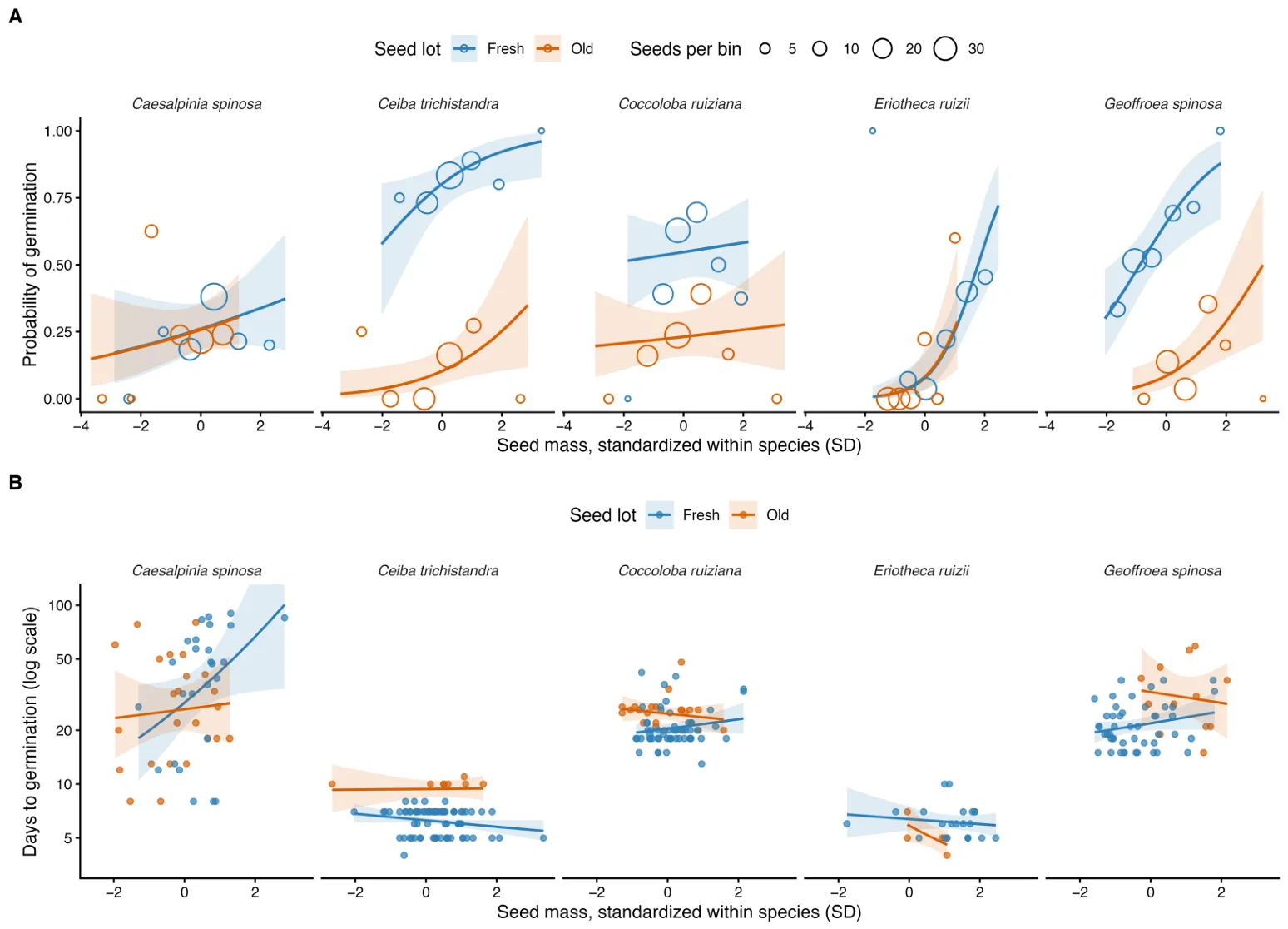
Seed mass and germination in five dry-forest tree species, historical (orange) versus fresh (blue) lots, with seed mass standardized within each species. (**A**) Fitted probability of germination from the retained binomial model with its 95% confidence band, drawn only over the range of seed mass observed in each species and lot; open circles are the observed proportion of seeds that germinated within equal-width bins of seed mass, with circle area proportional to the number of seeds in the bin. (**B**) Days to germination against seed mass among germinated seeds, with fitted Gamma regressions and their confidence bands. Hypothesis 3 concerns panel (**B**), where seed mass adds nothing to the model; panel (**A**) shows the route the hypothesis did not consider, in which heavier seeds are more likely to germinate by an amount that varies among species but does not differ between lots. Because panel (**B**) includes germinated seeds only, it compares fractions of each lot that may have been selected differently.

Heavier seeds were more likely to germinate, but the strength of that relationship varied among species and did not differ between lots. Adding a common seed-mass slope to the germination model improved the fit (Δdeviance = 21.3, d.f. = 1, *p* < 0.001), and allowing the slope to vary among species improved it further (Δdeviance = 19.4, d.f. = 4, *p* < 0.001; AIC 942.4 against 953.8). Allowing it to differ between the historical and the fresh lot, which is what Hypothesis 3 asserts, did not (Δdeviance = 6.5, d.f. = 5, *p* = 0.26; AIC 945.8; Table S3). Per standard deviation of within-species seed mass, the odds of germinating rose 4.01-fold in *E. ruizii* (2.15–7.48), 2.08-fold in *G. spinosa* (1.29–3.33) and 1.71-fold in *C. trichistandra* (1.07–2.73) and were indistinguishable from unchanged in *C. spinosa* and *C. ruiziana* (Figure 3A).

Seed mass did not predict germination speed at all. Adding it to the model of days to germination did not improve the fit (*p* = 0.57), nor did allowing its effect to vary by species and lot (*p* = 0.068; Figure 3B). Hypothesis 3 therefore finds no support, but the evidence differs between the two routes. Because days to germination exist only for germinated seeds, Figure 3B compares fractions of the historical and fresh lots that may have been selected differently, and the absence of a mass effect on germination time is weak evidence against buffering. Figure 3A, which includes every sown seed, supports only the narrower conclusion that the relationship between seed mass and the probability of germination did not differ detectably between historical and contemporary lots.

## 3. Discussion

### 3.1. Germination Capacity Retained by Historical Lots

This study provides, to our knowledge, the first assessment of germination capacity after 12 to 17 years of ambient laboratory storage for native Ecuadorian TDF species, extending the 36-month window examined by Silveira et al. [22] for *Mimosa foliolosa* by a factor of four to six and extending to a tropical dry-forest flora a body of evidence on ambient storage built almost entirely on temperate orthodox crop seeds [15,16]. Historical lots germinated less than fresh lots in four of the five species, with relative differences of 58 to 87%, and about as much as fresh lots in *C. spinosa*, so the first expectation holds for germination percentage. It holds less cleanly for germination speed. Three species germinated later from historical seed, and these were the three with the largest declines in germination percentage, so in them the probability and the speed of germination changed together, which is consistent with, although not proof of, the graded-damage model of seed aging [9,13,24]. The other two moved in the opposite direction: *C. spinosa* germinated 13 days earlier from historical seed and *E. ruizii* one day earlier, although neither contrast was clear after correction. In *C. spinosa*, a Fabaceae with a hard seed coat that received only imbibition as a pre-treatment, earlier germination from older seed is what one would expect if prolonged dry storage had weakened the coat, releasing physical dormancy rather than degrading the embryo [25,26]. Because the germinated fractions differ so much between lots, these timing differences may also reflect selective loss of slow-germinating seeds (Section 4.4).

Low germination in the fresh lots of *C. spinosa* (27.1%) and *E. ruizii* (22.9%) limits what their comparisons can show. Seeds of *C. spinosa* have the hard, water-impermeable coat associated with physical dormancy in Fabaceae [27], and our only pre-treatment was a 48 h soak, without scarification. Many ungerminated fresh seeds may therefore have been dormant rather than dead. The similar germination of the two lots cannot then be read as tolerance of storage: the historical lot may have lost viable seeds while others became germinable as the seed coat turned permeable during dry storage [25,26]. The difference attributable to storage is unknown in this species. A hard seed coat is not protective by itself: the other hard-seeded Fabaceae, *G. spinosa*, germinated 74.5% less in its historical lot. In *E. ruizii*, physical dormancy is less likely, because the confamilial *C. trichistandra* germinated at 81.2% under the same treatment. Low germination may instead reflect a high share of unfilled or immature seeds that visual inspection did not detect, or the short monitoring period (18 days) produced by our stopping rule; the 43% lower mass of the historical seeds also suggests that the two lots differed in seed fill. For both species, a viability test of ungerminated seeds and, for *C. spinosa*, a scarified control would be needed to separate dormancy, seed quality and storage.

Our observations are best read against studies that followed seed lots of known initial quality under defined conditions. For crop seeds kept under ambient conditions, longevity is usually limited and varies widely among species [15]. Even for orthodox seeds, survival over decades depends on species and, above all, on storage temperature and seed moisture content [16,24]. Against that background, the retention of 5 to 23% germination in four historical lots, and of germination similar to the fresh lot in *C. spinosa*, after 12 to 17 years in paper bags at room temperature is a modest but useful empirical result for tropical dry-forest trees, a group for which decade-scale storage data are scarce. It shows that ambient-stored institutional holdings retain some value for restoration, while remaining far from the conditions required for long-term conservation.

### 3.2. Storage Behavior of Coccoloba ruiziana

With a single species listed as recalcitrant [23], the second expectation could not be tested statistically: the effect of desiccation-tolerance class is inseparable from the identity of *C. ruiziana*, so the ranking we report is an observational comparison, not a formal test of class effects. The germination capacity retained by *C. ruiziana* is nevertheless difficult to reconcile with recalcitrant behavior. Recalcitrant seeds are shed at high moisture content and usually lose viability within weeks to months once dried [17,28], whereas 23 of the 100 seeds of the historical lot germinated after 14 years of dry storage in paper bags. This agrees with earlier work in which seeds of this species, dried over silica gel from 21.4% to 2% moisture content, kept germination above 50% (undried seeds germinated 32%), and which classified the species as intermediate rather than recalcitrant [26]. Storage behavior varies within the genus: seeds of the Amazonian *C. gigantifolia* are desiccation-sensitive [29], whereas seeds of *C. uvifera* kept high germination after 15 months at room temperature [30]. Desiccation tolerance together with retention of germination over more than a decade is inconsistent with recalcitrant behavior and consistent with intermediate or orthodox behavior in *C. ruiziana*, and we suggest that its regional classification [23] be revisited. A definitive classification still requires viability–moisture-content curves and longevity tests at contrasting moisture contents and temperatures [17,24]. The ranking of species by relative difference followed the number of years each lot had been held more closely than desiccation-tolerance class (Figure S3), but duration, species identity and collection year are fully confounded in our set. The ranking among species is further confounded with sowing date, because the five species were sown in two groups (March and May 2023) owing to greenhouse space; comparisons between lots within a species are not affected, because both lots of each species were sown together. The pattern is therefore a hypothesis for a design with lots of known age from a single collection event (Section 3.6).

### 3.3. Seed Mass and Germination

Hypothesis 3 received no support by either of the two routes through which larger seeds might buffer storage. Seed mass was unrelated to germination speed among germinated seeds, but this is weak evidence, because those seeds may be differently selected fractions of the historical and fresh lots (Section 4.4). It did predict whether a seed germinated at all, and by an amount that differed among species, from a fourfold increase in the odds per standard deviation in *E. ruizii* to no detectable effect in *C. spinosa* and *C. ruiziana*. That effect did not differ detectably between historical and fresh lots, which buffering would require; within a species, heavier seeds were more likely to germinate in both lots [20,21]. The question of which traits do govern post-storage behavior remains open. That variation followed neither family nor seed-coat type: the two hard-coated Fabaceae fell at opposite ends, with seed mass predicting germination in *G. spinosa* but not in *C. spinosa.* The relevant differences therefore lie in traits we did not measure. Direct tests of seed-coat permeability and dormancy class [27] would be the logical next step, particularly for *C. spinosa*, whose germination held up, but which was also the most recently collected of the five lots.

### 3.4. Seedling Growth

Whether differences between lots extend beyond germination has a mixed answer. Only *C. ruiziana* showed a carry-over that survives scrutiny: its historical seed produced seedlings 1.31 cm shorter, and the deficit persisted, at 0.80 cm, once seedling age at the census was accounted for. In the other four species, the contrasts were either unclear after correction, as in *E. ruizii*, whose historical-lot seedlings were 2.03 cm shorter at Holm *p* = 0.104, or ran in favor of the historical lot: seedlings were 2.25 cm taller and mean relative growth rate was 4.6 times higher in the historical lot of *C. trichistandra* and 1.8 times higher in that of *E. ruizii*. Selection is the parsimonious reading of those two cases: where only one seed in ten or twenty germinates, the seedlings that appear are those whose seeds retained the capacity to germinate, so faster growth among them reflects the severity of the filter rather than a benefit of storage. Post-germination consequences of ambient storage may well exist, but detecting them requires a design that separates the cohort from the treatment, for instance by comparing seedlings matched on emergence date. For restoration planning, the implication is narrower than the germination result: the main difference between historical and fresh lots lies in how many seeds germinate, not the vigor of the seedlings that do.

### 3.5. Implications for Restoration and Existing Seed Collections

What a nursery needs is the number of seedlings obtained per unit of seed sown, and at the germination stage alone the contrast is already large. Per 100 seeds sown, historical lots germinated 10.4 against 81.2 for fresh seed in *C. trichistandra*, 23.0 against 55.0 in *C. ruiziana*, 14.0 against 54.7 in *G. spinosa* and 5.2 against 22.9 in *E. ruizii,* while *C. spinosa* differed by 2.1 percentage points, 25.0 against 27.1. These values describe the particular lots we tested and cannot be transferred to other lots or years. They indicate, nonetheless, that 12- to 17-year-old ambient holdings can still yield seedlings but should not be assumed equivalent to fresh seed: managers should run a germination test on each holding before planning sowing quantities, expect to sow several times more seed per seedling produced [8,31,32], and budget longer and less synchronous production. These germination shortfalls fall within the range of seed wastage reported for seed-based restoration more generally [33].

### 3.6. Limitations and Recommendations for Future Studies

Several limitations qualify these results. Historical and fresh lots came from different collection years and from different sets of mother trees; seeds were pooled by species and maternal identity was not retained, so the mother tree rather than the seed is the biological replicate, and our confidence intervals understate the uncertainty attached to historical lots in general. No germination test was made on the historical lots at collection, so each comparison describes retained germination capacity relative to a contemporary lot, not a loss caused by storage. We sowed in two groups because the greenhouse space available to us could not accommodate all containers at once, which leaves comparisons among species confounded with sowing group, although comparisons between lots within a species are unaffected. We ran no viability test on ungerminated seeds, so we cannot distinguish dead from dormant, a distinction that matters most for the two hard-coated Fabaceae. Final size and growth rate are interim values at each group’s last census, day 87 or day 95, rather than end-of-trial values at day 108, since the data available covered 23 of 29 planned censuses. They are also truncated for part of the sample: monitoring ended early for individuals that were removed at intervals for destructive measurement of root and leaf traits reported elsewhere, and because the records do not identify which individuals were removed, we cannot separate removals from genuine mortality. We therefore do not analyze seedling survival, and we report height and growth rate up to each individual’s last measurement. Temperature, relative humidity and seed moisture content were not recorded during storage, precluding any estimate of aging kinetics [13,24], and formal desiccation-tolerance testing is still needed for all five species.

A design able to estimate the effect of storage, rather than the germination capacity retained by historical lots, would require: (1) a single, dated collection per species, with maternal identity recorded, divided into subsamples tested at collection and at fixed intervals, so that each lot serves as its own baseline; (2) seed moisture content measured at collection, after drying and at each test, with storage temperature and relative humidity logged continuously; (3) contrasting storage treatments, such as ambient laboratory storage, hermetic storage after drying, and cold storage, so that longevity can be modeled with the viability equation [24]; (4) germination tests in replicated subsamples, for example four replicates of 25 seeds, arranged in randomized blocks, with species-appropriate dormancy-breaking pre-treatments such as scarification for hard-seeded Fabaceae, and a fixed test period long enough for slow species; (5) a tetrazolium or cut test of every ungerminated seed, to separate dead from dormant seeds; (6) direct tests of desiccation tolerance for each species, particularly *C. ruiziana*; and (7) seedling comparisons matched on emergence date, with individuals removed for destructive sampling recorded individually.

## 4. Materials and Methods

### 4.1. Study Species and Site

We studied five woody species representative of TDF communities of the Zapotillo, Macará and Célica cantons in Loja Province, southern Ecuador (3–4° S, 79–80° W; 400–600 m a.s.l.), where all seed lots used here were collected. We selected them because they are common canopy and sub-canopy trees of these communities, are routinely used in local restoration and nursery programs, and were the species for which both a historical and a freshly collected lot were available. Mean annual temperature is approximately 27 °C; monthly rainfall is below 10 mm in the dry season, from June to November, and reaches 200 mm in the wet season, from December to May [34,35].

The five species differ in fruit type and seed structure. *Caesalpinia spinosa* (Mol.) Kuntze (accepted name *Tara spinosa* (Molina) Britton & Rose [36]) and *Geoffroea spinosa* Jacq. (Fabaceae) produce indehiscent fruits with hard-coated seeds, pods in the former and large drupes in the latter; *Ceiba trichistandra* (A.Gray) Bakh. and *Eriotheca ruizii* (K.Schum.) A.Robyns (Malvaceae) produce capsules with small seeds embedded in cottony material; and *Coccoloba ruiziana* Lindau (Polygonaceae) produces fleshy achenes. Romero-Saritama and Draper [23] list *C. ruiziana* as recalcitrant and the other four species as orthodox; we treat *C. ruiziana* as intermediate following [37] (Table 1, Section 3.2). That classification comes from a regional compilation rather than from species-specific moisture-content viability curves, and no species in our set has been classified following the protocols of Berjak and Pammenter [17]; we therefore treat desiccation-tolerance class throughout as a working assumption rather than a measured trait.

**Table 1:** Study species, life form, presumed desiccation-tolerance class, age of the historical lot, number of seeds measured per lot, and sowing group. Years in storage are counted from the collection year of the historical lot to the date of sowing. Desiccation class follows the regional compilation [23], except *C. ruiziana*, listed there as recalcitrant but treated here as intermediate following [37] (Section 3.2). The numbers of seeds sown and germinated per lot are given in Figure 4.

Species	Life Form	Desiccation Class	*n* Measured (Historical/Fresh)	Historical-Lot Collection Year/Years in Storage	Sowing Group (Date)
*Caesalpinia spinosa*	Medium tree	Orthodox	100/100	2011/12	25 May 2023
*Ceiba trichistandra*	Large canopy tree	Orthodox	100/100	2006/17	17 March 2023
*Coccoloba ruiziana*	Small tree/shrub	Intermediate	100/100	2009/14	25 May 2023
*Eriotheca ruizii*	Small tree	Orthodox	100/100	2008/15	17 March 2023
*Geoffroea spinosa*	Tree (large drupes)	Orthodox	100/86	2008/15	17 March 2023

### 4.2. Seed Lots

For the fresh lots, we collected seeds from 10 adult individuals per species and 10 fruits per individual and pooled them by species. Collection took place at the end of the fruiting season of each species, and we sowed each lot as soon as enough cleaned seed was available. The historical lots had been collected following the same protocol, from 10 individuals per species, but not from the same trees as the fresh lots. Because seeds were pooled by species in both cases and mother-tree identity was not retained, maternal variation could not be controlled for in either lot.

The historical lots comprised seeds collected between 2006 and 2011 and held under ambient laboratory conditions in the Plant Ecophysiology Laboratory, UTPL, accumulating 12 to 17 years of storage by the time they were sown (Table 1). Storage was not controlled: seeds were kept in closed paper bags at a room temperature of approximately 18 to 24 °C (Plant Ecophysiology Laboratory staff, UTPL, personal communication), with no regulation of relative humidity. Neither temperature nor relative humidity was logged during storage, and seed moisture content was not recorded at collection or at sowing. Because temperature and seed moisture content are the main determinants of the rate of seed aging [24], the storage history of these lots is known only approximately, and our data cannot be used to estimate aging kinetics or to attribute differences to particular storage conditions. These conditions also differ from genebank storage, in which orthodox seeds are dried in equilibrium with 10–25% relative humidity and held at about −18 °C [38]. We did not measure moisture content at sowing because it would have reflected only equilibrium with laboratory air at that time rather than the moisture history of the lots, and because the oven method is destructive, and every available seed was needed for the germination test. No germination or viability test was made on the historical lots at collection, so their initial germination capacity is unknown. Historical and fresh lots therefore came from different years, and from different sets of mother trees. Any difference between historical and fresh lots reported below confounds storage duration with inter-annual variation in initial seed quality, so we report these differences as lot-level associations rather than as isolable storage effects.

We coded seeds individually, using codes 1 to 100 for the historical lot and 101 to 200 for the fresh lot. We cleaned the seeds of pulp or cottony material, inspected them visually, and discarded those with visible damage such as holes or a broken seed coat, as well as those whose flattened outline indicated an empty seed. We then soaked all seeds in distilled water for 48 h before sowing; this was the sole pre-germination treatment, with no scarification, stratification or hormonal treatment.

### 4.3. Seed Measurements

Before imbibition, we measured each individually coded seed: mass (g) on an analytical balance (precision 0.0001 g; AGZN220 Torbal, Bohemia, NY, USA), and length and width (mm) from calibrated digital scans (Plustek OpticPro A320E; Plustek Inc., Taipei, Taiwan, with an individually numbered reference grid) analyzed in ImageJ v.1.53. We took length as the longest axis of the seed and width as the longest axis perpendicular to it. Seeds retained their code through sowing, so every measured seed can be matched to its germination record. We measured 986 seeds in total, slightly more than the 948 we sowed; sample sizes per species and lot are given in Table 1.

### 4.4. Germination Experiment

We sowed seeds individually in labeled containers filled with peat substrate under greenhouse conditions at the Ecophysiology Laboratory, UTPL (Loja, 2100 m a.s.l., ambient temperature 18 to 24 °C and natural photoperiod of approximately 12 h). We sowed in two groups: *C. trichistandra*, *E. ruizii* and *G. spinosa* on 17 March 2023, and *C. spinosa* and *C. ruiziana* on 25 May 2023, due to space availability in our greenhouse. Containers were arranged in blocks on greenhouse shelves. Within each species, we sowed the same number of seeds in each lot, drawn at random from the 100 coded seeds of that lot: 96 for *C. spinosa*, *C. trichistandra* and *E. ruizii,* 100 for *C. ruiziana* and 86 for *G. spinosa*, numbers that reflect the seed quantity available. We recorded germination daily, taking radicle emergence of at least 2 mm as the criterion, and continued until no new germination occurred over seven consecutive days; monitoring therefore lasted from 18 days in *E. ruizii* to 97 days in *C. spinosa*, whose last seed germinated on day 90.

We defined germination percentage (GP) as the number of germinated seeds divided by the number sown so that ungerminated seeds entered the denominator. We calculated mean germination time (MGT, days) as Σ(n_i_t_i_)/Σ(n_i_), where n_i_ is the number of seeds germinating on day t_i_ [18], and the germination speed index (GSI) as Σ(n_i_/t_i_) [19]. GSI measures germination speed and vigor rather than synchrony, and because it does not normalize for the number of seeds sown, it also rises with the number of germinants. We estimated T50g as the median germination day among germinated seeds; we did not use the time for 50% of sown seeds to germinate, because it is undefined in the seven lots whose final germination was below 50%. MGT, GSI and T50g are computed on germinated seeds only, so they describe different fractions of each lot when final germination differs. A shorter MGT in a lot with low GP may reflect faster germination, but it may equally reflect the absence of a slow-germinating fraction that failed to germinate. We therefore interpret these indices only alongside GP and the time-to-event analysis (Section 2.6). Ungerminated seeds were not tested for viability at the end of monitoring, for example by tetrazolium or cut test. GP therefore measures germination capacity under our test conditions: an ungerminated seed may have been dead, but it may also have been dormant, physically unable to imbibe, or lacking a cue that the test did not supply.

### 4.5. Seedling Monitoring

We recorded shoot height (cm) and leaf number at 29 census occasions per sowing group, at intervals of 3 to 4 days, covering the first 108 days since that group was sown. The data analyzed here cover the first 23 censuses of each group: 27 March to 12 June 2023 for the first group, days 10 to 87 since sowing, and 12 June to 28 August 2023 for the second, days 18 to 95. Because the census intervals are identical in the two groups, growth rates are comparable across species; because the series start at different numbers of days after sowing, we report final size at each group’s last census rather than at a common date. These are interim rather than end-of-trial values. We watered the containers every four days throughout the experiment.

We calculated relative height growth rate (RGR, cm cm^−1^ day^−1^) as (ln H_i+1_ − ln H_i_)/(t_i+1_ − t_i_) [39]. We read a height of zero before an individual’s first positive height as pre-emergence and excluded it from RGR; where a height of zero followed a positive height and remained at zero thereafter, we treated the last positive census as that individual’s last measurement and computed no further growth increments. Four seeds germinated but never produced a measurable shoot: we counted them as germinated in GP and excluded them from height, leaf number and RGR.

### 4.6. Statistical Analysis

**General approach.** We performed all analyses in R 4.4.1 (R Foundation for Statistical Computing, Vienna, Austria) [40], fitting generalized linear models with glmmTMB 1.1.10 [41], obtaining type II tests with car 3.1-5 [42], estimating marginal means and contrasts with emmeans 1.11.2 [43], validating every model with scaled quantile residuals in DHARMa 0.4.7 [44]. No response variable was normally distributed (Shapiro–Wilk *p* < 0.001 in all cases), so we selected each error family by AIC among those admissible for its measurement scale, guided by the log-variance-to-log-mean relationship across the ten species × lot cells. Because the five species differ by three orders of magnitude in seed mass and by more than two in residual variance, we let the dispersion parameter vary among species; a single pooled dispersion was rejected by residual diagnostics in every continuous response. All models reported here passed tests of residual uniformity, dispersion and outliers. All tests were two-tailed, and within each family of five per-species contrasts we corrected *p* values with the Holm–Bonferroni procedure, reporting both values. Where one lot contributed fewer than ten individuals to a contrast, we also report the exact Mann–Whitney test and give it precedence where the two disagree, because asymptotic uncertainty is unreliable at that sample size.

**Units of inference.** Each species × lot combination is a single, non-replicated sown batch, so batch-level overdispersion is not estimable, and the individual seed is the unit of inference. Seeds were pooled by species and mother-tree identity was not retained, so the mother tree rather than the seed is the biological replicate; confidence intervals therefore describe variation among seeds within the batches tested and understate the uncertainty attached to historical lots in general. Contrasts between the historical and the fresh lot of a given species were internally consistent because both lots shared a sowing date and census schedule; comparisons among species are confounded with sowing group.

**Seed-lot characterization.** We modeled seed mass, length and width (n = 986) with Gamma models with log link and species-specific dispersion, fitting species, lot and their interaction, and expressed historical-versus-fresh contrasts as ratios. These contrasts characterize the lots as received; we cannot resolve them into causes because a lot difference in seed size may reflect the mother trees and crop year each lot came from, or desiccation and reserve catabolism during storage. Seed size is therefore a potential confounder and a potential consequence of storage at once, so we did not adjust the germination models for it: adjusting for a variable that may lie on the causal path from storage to germination would remove part of the effect under study.

**Hypothesis** **1.** *We modeled germination as a Bernoulli response of the individual seed (n = 948) with a binomial model with logit link, including species, lot and their interaction, and tested terms by likelihood-ratio chi-square, expressing contrasts as odds ratios of the historical relative to the fresh lot. Days to germination are a positive, right-skewed count with a variance-to-mean ratio of 13.6, so we modeled them with a Gamma model with log link and species-specific dispersion (n = 302) rather than by analysis of variance; AIC preferred this over Poisson, negative-binomial and constant-dispersion Gamma alternatives, and it was the only specification whose residuals passed all three diagnostics. We expressed contrasts as ratios of mean germination times and interpreted the lot term only alongside the interaction, because a main effect averages over species that respond in opposite directions. Because germination day exists only for seeds that germinated, these analyses compare lots whose germination fractions differ by up to eightfold; we therefore also modeled germination as a time-to-event process over all 948 sown seeds, using an accelerated failure time model with lognormal errors fitted in survival 3.6-4 [45], treating each germination as having occurred between consecutive censuses and ungerminated seeds as right-censored at the end of their group’s monitoring. This combines how many seeds germinate and how fast into one analysis that is not conditional on having germinated. We treat the time-to-event analysis as the primary analysis of germination timing, because it uses every sown seed, and the Gamma model as a secondary description of timing among germinated seeds only; per-species time ratios from the time-to-event model are given in Table S4. For Table 2 we report, alongside the model-based odds ratios, p values from Fisher’s exact test (germination) and from the Mann–Whitney test (days to germination), Holm-corrected across the five species.*

**Table 2:** Germination timing of five dry-forest tree species from historical (12–17 years of ambient laboratory storage) and freshly collected seed lots. Germination percentages, odds ratios and their *p* values are given in Figure 1 and Section 2.2, and the numbers of seeds sown and germinated per lot in Figure 1. MGT, GSI and T50g were calculated on the germinated seeds only; these indices describe germination timing among germinants only and are not comparable between lots as measures of overall vigor when final germination differs strongly (Section 4.4). MGT, mean germination time; GSI, germination speed index, which also rises with the number of germinants; T50g, median germination day among germinants. *p* (MGT) is from the Mann–Whitney test, Holm-corrected across the five species. Time ratios for MGT from the Gamma model are given in Section 2.4, and those from the time-to-event model, which uses all sown seeds, in Table S4.

Species	Lot	MGT (Days)	GSI	T50g (d)	*p* (MGT)
*Caesalpinia spinosa*	Historical	31.9	1.15	24.5	0.19
	Fresh	45.2	1.05	47.5	
*Ceiba trichistandra*	Historical	9.5	1.08	10.0	<0.001
	Fresh	6.3	12.76	7.0	
*Coccoloba ruiziana*	Historical	25.4	0.94	26.0	<0.001
	Fresh	21.3	2.74	20.0	
*Eriotheca ruizii*	Historical	5.2	0.99	5.0	0.19
	Fresh	6.3	3.66	6.0	
*Geoffroea spinosa*	Historical	33.3	0.42	29.5	0.016
	Fresh	22.2	2.30	21.0

**Hypothesis** **2.** *With a single species listed as recalcitrant [23], we cannot test desiccation-tolerance class as a factor: its effect is inseparable from the identity of C. ruiziana. We therefore evaluated Hypothesis 2 qualitatively, ranking the five species by relative loss in germination percentage and asking whether that species occupied the extreme of that ranking. Because it fell in the middle, we then examined the most obvious alternative predictor available, the number of years each historical lot had been held, which we describe with Spearman’s rank correlation coefficient, without a significance test. The unit here is the species, so n = 5; the species are not independent replicates, and each species also differs in collection year, set of mother trees and, in part, sowing group; we present this comparison as exploratory, to generate a hypothesis for a future design, not as a test.*

**Hypothesis** **3.** *Hypothesis 3 asserts that the effect of seed mass differs between lots, which requires a lot-specific slope rather than merely a mass effect. We standardized seed mass within species and fitted four nested binomial models of germination: species × lot with no mass term, a common slope, a slope specific to each species, and a slope specific to each species and lot, comparing them by likelihood-ratio tests and AIC. We repeated the sequence on the Gamma model of days to germination, the route the hypothesis names explicitly. We did not retain the saturated model, whose lot-specific term was significant only when E. ruizii was included: with five of 96 historical seeds germinating and the heaviest germinants lying above every ungerminated seed, its slope is a near-separation artifact with a confidence interval spanning two orders of magnitude. We matched seeds between the morphometric and germination datasets by their unique code, with an unambiguous match for all 302 germinated individuals.*

## 5. Conclusions

Historical seed lots of five native Ecuadorian dry-forest tree species, kept for 12 to 17 years under uncontrolled ambient laboratory conditions, still germinated, although less than freshly collected lots in four of the five species (relative differences of 58 to 87%). Because the lots were independent collections and ungerminated seeds were not tested for viability, these differences describe retained germination capacity, not the effect of storage alone. In *C. spinosa*, the two lots germinated similarly, but low germination in both may reflect physical dormancy. Germination percentage and germination timing did not change in parallel across the five species. *Coccoloba ruiziana* retained 23% germination after 14 years of dry storage, which, together with earlier evidence of desiccation tolerance, argues against its classification as recalcitrant and calls for direct tests of its storage behavior. Only in *C. ruiziana* did historical seed produce clearly shorter seedlings. Ambient-stored institutional holdings of these species can still supply seedlings for restoration, but each holding should be tested before use, sowing quantities must be species-specific, and *C. trichistandra*, *G. spinosa*, *C. ruiziana* and *E. ruizii* are priorities for renewed collection under controlled storage.

## Figures and Tables

**Figure 4:**
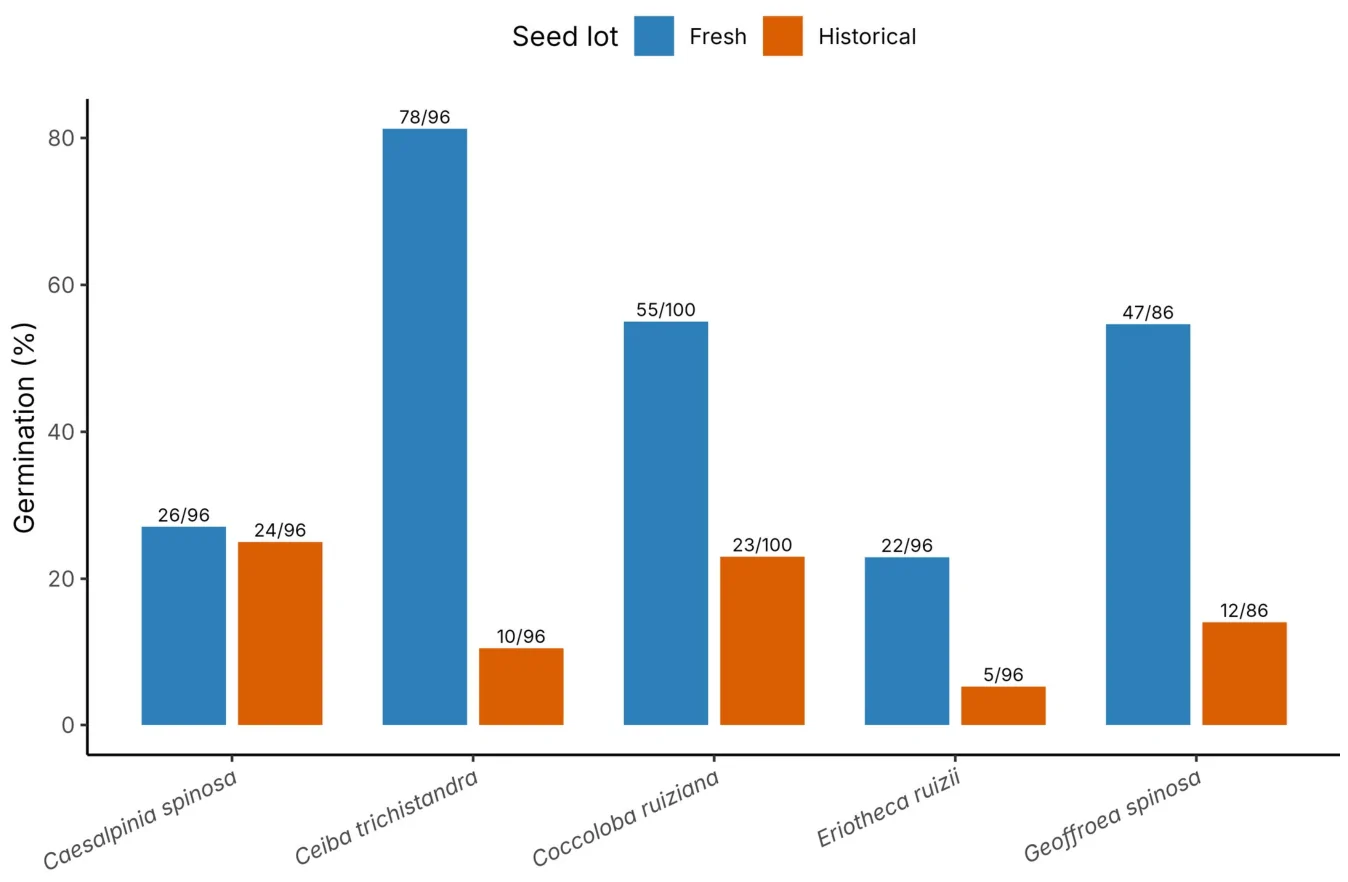
Germination percentage of five dry-forest tree species from historical (orange) and freshly collected (blue) seed lots. Numbers above each bar are germinated seeds over seeds sown. Ungerminated seeds are included in the denominator.

## Data Availability

The seed morphometric, germination and seedling growth data supporting the results reported in this article are openly available in Figshare at https://doi.org/10.6084/m9.figshare.34004151.

## Supplementary Materials

The following supporting information can be downloaded at https://www.mdpi.com/article/10.3390/plants15213381/s1, Figure S1: Seed mass (A), length (B) and width (C) of the historical (orange) and fresh (blue) lots of five dry-forest tree species (all 986 measured seeds); Figure S2: Final shoot height (A) and number of leaves (B) of seedlings from the historical (orange) and fresh (blue) lots, at each individual’s last measurement; Figure S3: Exploratory comparison of the relative difference in germination between the historical and the fresh lot (as a percentage of the fresh-lot value) and the number of years each historical lot had been held under ambient laboratory conditions at sowing (collection year in parentheses); Table S1: Seed size of the historical and fresh lots; Table S2: Seedling growth of the historical and fresh lots, recorded at each individual’s last measurement (interim values at day 87 or 95 since sowing); Table S3: Seed mass and the probability of germination (n = 948 sown seeds; seed mass standardized within species); Table S4: Time-to-event (accelerated failure time) model of germination over all 948 sown seeds: estimated median time to germination by lot and time ratio of the historical relative to the fresh lot, per species. The seed morphometric, germination and seedling growth data supporting the results reported in this article are openly available in Figshare at https://doi.org/10.6084/m9.figshare.34004151. The supplementary tables and figures are also archived in Zenodo at https://doi.org/10.5281/zenodo.23019272. The supplementary materials include Figure S3, an exploratory comparison of relative germination difference and years in storage, and Table S4, per-species time ratios from the time-to-event model. The R code that reproduces every analysis, table and figure is available from the corresponding author on request.
