# Supplementary material for: Germination Capacity of Five Dry-Forest Tree Species from Southern Ecuador After 12 to 17 Years of Ambient Laboratory Storage
Source: Plants (Basel). 2026 Nov 1;15(21):3381. doi: 10.3390/plants15213381 (PMC13765987; doi:10.3390/plants15213381)
Supplement: Supplementary file 1 [file plants-15-03381-s001.zip › plants-4626170-supplementary.pdf]

Supplementary Materials

Germination capacity of five dry-forest tree species from southern Ecuador after 12 to 17 years of ambient laboratory storage

Cañar J., Vélez-Mora D.P., Gusmán-Montalván E. — *Plants*, manuscript plants-4626170 (revised version). In all tables, “Historical” denotes the lots stored 12–17 years under ambient laboratory conditions and “Fresh” the lots collected for this study; contrasts are historical relative to fresh, and P values are Holm-corrected across the five species.

Table S1. Seed size of the historical and fresh lots (all 986 measured seeds: 100 per lot, except 86 in the fresh lot of *Geoffroea spinosa*). Ratios from Gamma models with log link and species-specific dispersion (seed trait ~ species × lot). Because the lots come from different collection years and mother trees, these contrasts describe the lots as received and cannot be attributed to storage (Section 3.1).

**Species**	**Trait**	**Fresh, mean (SD)**	**Historical, mean (SD)**	**Ratio [95 % CI]**	**Holm P**
Caesalpinia spinosa	Seed mass (g)	0.260 (0.030)	0.245 (0.030)	0.945 [0.913–0.978]	0.004
	Seed length (mm)	13.08 (0.64)	11.45 (0.83)	0.875 [0.860–0.890]	< 0.001
	Seed width (mm)	8.84 (0.63)	8.99 (0.68)	1.017 [0.996–1.037]	0.115
Ceiba trichistandra	Seed mass (g)	0.214 (0.022)	0.204 (0.026)	0.953 [0.922–0.986]	0.010
	Seed length (mm)	10.30 (0.47)	11.16 (0.77)	1.083 [1.066–1.101]	< 0.001
	Seed width (mm)	9.01 (0.59)	8.67 (0.71)	0.962 [0.942–0.982]	< 0.001
Coccoloba ruiziana	Seed mass (g)	0.044 (0.007)	0.042 (0.010)	0.950 [0.900–1.003]	0.064
	Seed length (mm)	5.18 (0.34)	5.20 (0.51)	1.003 [0.982–1.025]	0.766
	Seed width (mm)	4.72 (0.38)	4.62 (0.38)	0.979 [0.957–1.001]	0.115
Eriotheca ruizii	Seed mass (g)	0.045 (0.013)	0.026 (0.009)	0.570 [0.520–0.624]	< 0.001
	Seed length (mm)	6.83 (0.44)	6.02 (0.46)	0.883 [0.866–0.900]	< 0.001
	Seed width (mm)	5.07 (0.34)	4.39 (0.36)	0.865 [0.848–0.883]	< 0.001
Geoffroea spinosa	Seed mass (g)	3.884 (1.102)	5.358 (1.046)	1.380 [1.289–1.476]	< 0.001
	Seed length (mm)	32.69 (3.93)	34.95 (3.09)	1.069 [1.038–1.101]	< 0.001
	Seed width (mm)	23.29 (2.54)	26.33 (2.04)	1.131 [1.101–1.161]	< 0.001

Table S2. Seedling growth of the historical and fresh lots, recorded at each individual's last measurement (interim values at day 87 or 95 since sowing). (A) Gaussian model with species-specific dispersion; (B) the same model with seedling age at the census as a covariate; (C) generalized Poisson model; (D) Gaussian model of log mean RGR, back-transformed to a ratio. Relative growth rate requires at least two positive height measurements. Contrasts based on fewer than ten seedlings in one lot (*E. ruizii*, *C. trichistandra* historical) should be read with the exact Mann–Whitney tests reported in Section 3.5; the RGR and leaf-number differences in the two most severely filtered lots are consistent with selection rather than with a benefit of storage (Section 3.5).

Species	n (fresh / historical)	Estimate [95 % CI]	Holm P
**A. Final shoot height: difference historical − fresh (cm)**			
Caesalpinia spinosa	25 / 24	0.30 [-0.66 to 1.26]	1.000
Ceiba trichistandra	78 / 10	2.25 [0.42 to 4.08]	0.064
Coccoloba ruiziana	54 / 22	-1.31 [-2.02 to -0.60]	0.002
Eriotheca ruizii	22 / 5	-2.03 [-3.91 to -0.15]	0.104
Geoffroea spinosa	46 / 12	-0.32 [-2.76 to 2.13]	1.000
**B. Final shoot height adjusted for seedling age: difference historical − fresh (cm)**			
Caesalpinia spinosa	25 / 24	-0.11 [-0.92 to 0.70]	1.000
Ceiba trichistandra	78 / 10	3.03 [1.00 to 5.06]	0.018
Coccoloba ruiziana	54 / 22	-0.80 [-1.33 to -0.26]	0.018
Eriotheca ruizii	22 / 5	-1.50 [-3.21 to 0.22]	0.260
Geoffroea spinosa	46 / 12	0.33 [-2.17 to 2.83]	1.000
**C. Leaf number: ratio historical/fresh**			
Caesalpinia spinosa	25 / 24	1.00 [0.86–1.17]	1.000
Ceiba trichistandra	78 / 10	0.95 [0.77–1.18]	1.000
Coccoloba ruiziana	54 / 22	0.76 [0.57–1.01]	0.177
Eriotheca ruizii	22 / 5	2.10 [1.47–3.00]	< 0.001
Geoffroea spinosa	46 / 12	1.19 [1.03–1.38]	0.082
**D. Mean relative growth rate: ratio historical/fresh**			
Caesalpinia spinosa	24 / 24	0.91 [0.67–1.23]	1.000
Ceiba trichistandra	78 / 10	2.58 [1.95–3.42]	< 0.001
Coccoloba ruiziana	53 / 21	1.06 [0.89–1.27]	1.000
Eriotheca ruizii	22 / 4	1.81 [1.18–2.78]	0.027
Geoffroea spinosa	46 / 12	1.09 [0.75–1.60]	1.000

Table S3. Seed mass and the probability of germination (n = 948 sown seeds; seed mass standardized within species). (A) Nested binomial models compared by likelihood-ratio tests, each against the model above it. The model with a species-specific mass slope common to both lots (lowest AIC) was retained; allowing the slope to differ between lots, which is what Hypothesis 3 requires, did not improve the fit. (B) Species-specific slopes from the retained model, expressed as the change in the odds of germinating per standard deviation of seed mass (P values not corrected).

(A) Model comparison

Binomial model of germination	Parameters	Deviance	ΔDeviance	P (LRT)	AIC
species × lot (no mass)	10	953.0	—	—	973.0
+ common mass slope	11	931.8	21.3	< 0.001	953.8
+ species-specific mass slope	15	912.4	19.4	< 0.001	942.4
+ species- and lot-specific mass slope	20	905.8	6.5	0.257	945.8

(B) Species-specific mass slopes in the retained model

Species	Odds ratio per SD of seed mass [95 % CI]	P
Caesalpinia spinosa	1.20 [0.85–1.71]	0.298
Ceiba trichistandra	1.71 [1.07–2.73]	0.025
Coccoloba ruiziana	1.07 [0.79–1.47]	0.658
Eriotheca ruizii	4.01 [2.15–7.48]	< 0.001
Geoffroea spinosa	2.08 [1.29–3.33]	0.002

Table S4. Time-to-event (accelerated failure time) model of germination over all 948 sown seeds: estimated median time to germination by lot and time ratio of the historical relative to the fresh lot, per species. Lognormal errors; germination interval-censored between consecutive daily records and ungerminated seeds right-censored; model germination ~ species × lot (survival package). Time ratios > 1 mean that the historical lot took longer to germinate once ungerminated seeds are taken into account; they combine how many seeds germinated and how fast. Medians of lots in which fewer than half of the seeds germinated (all historical lots and the fresh lots of *C. spinosa* and *E. ruizii*) lie beyond the observation period and are model extrapolations; the time ratio is the more robust summary.

**Species**	**Median, fresh (days)**	**Median, historical (days)**	**Time ratio [95 % CI]**	**Holm P**
Caesalpinia spinosa	137.2	129.4	0.94 [0.68–1.32]	0.732
Ceiba trichistandra	7.2	39.2	5.42 [3.66–8.04]	< 0.001
Coccoloba ruiziana	40.8	92.4	2.26 [1.64–3.12]	< 0.001
Eriotheca ruizii	20.1	43.5	2.17 [1.37–3.42]	0.002
Geoffroea spinosa	45.2	156.0	3.45 [2.37–5.03]	< 0.001

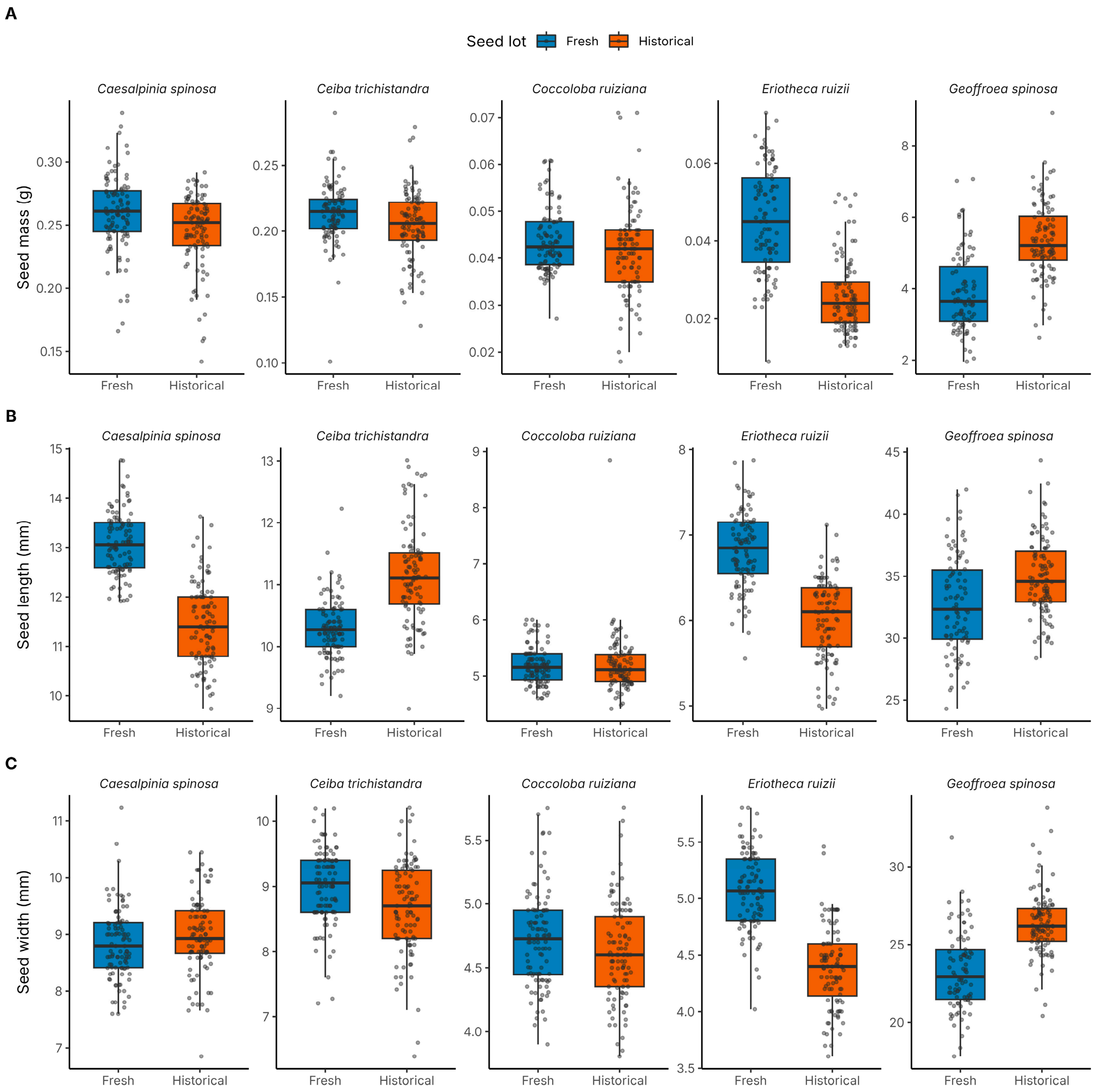

Figure S1. Seed mass (A), length (B) and width (C) of the historical (orange) and fresh (blue) lots of five dry-forest tree species (all 986 measured seeds). Boxes show the median and interquartile range, whiskers extend to 1.5 times the interquartile range, and grey points are individual seeds. Contrasts are given in Table S1; differences between lots describe the lots as received and cannot be attributed to storage.

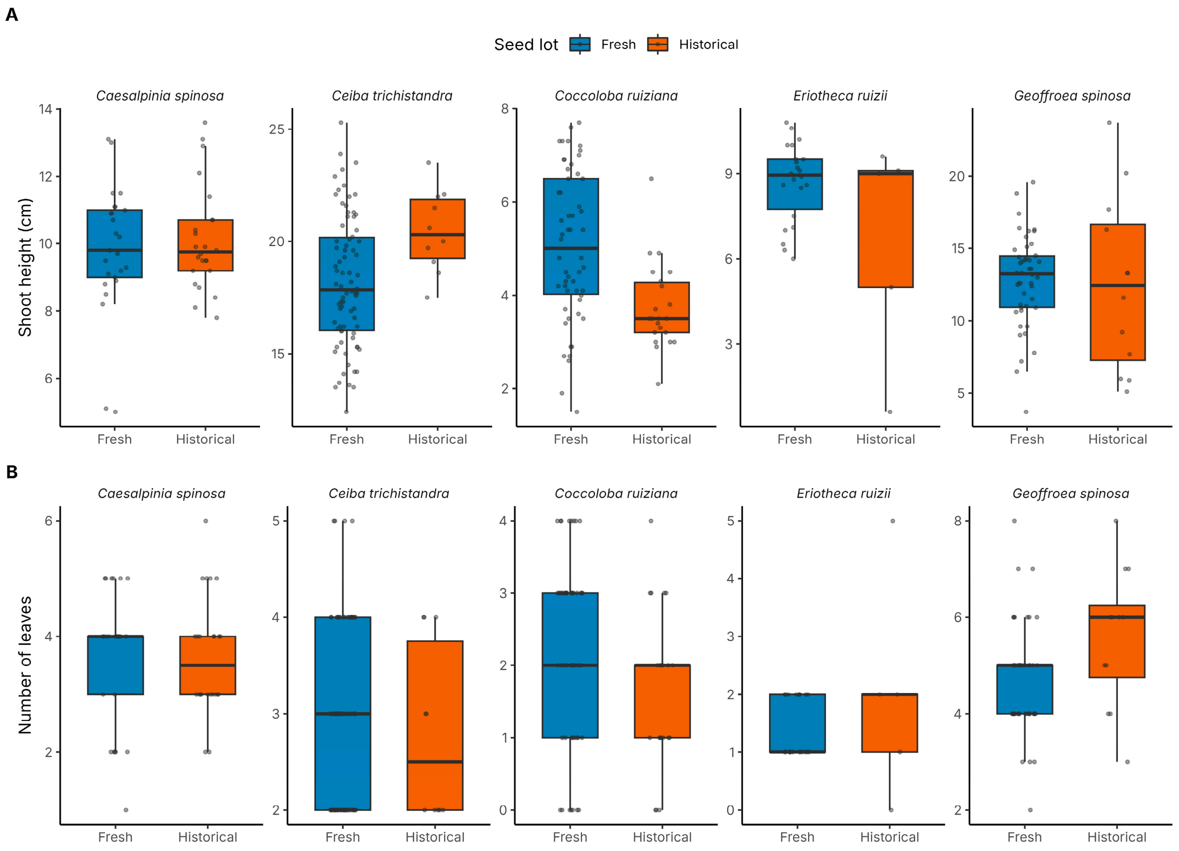

Figure S2. Final shoot height (A) and number of leaves (B) of seedlings from the historical (orange) and fresh (blue) lots, at each individual's last measurement. Boxes as in Figure S1. Contrasts are given in Table S2.

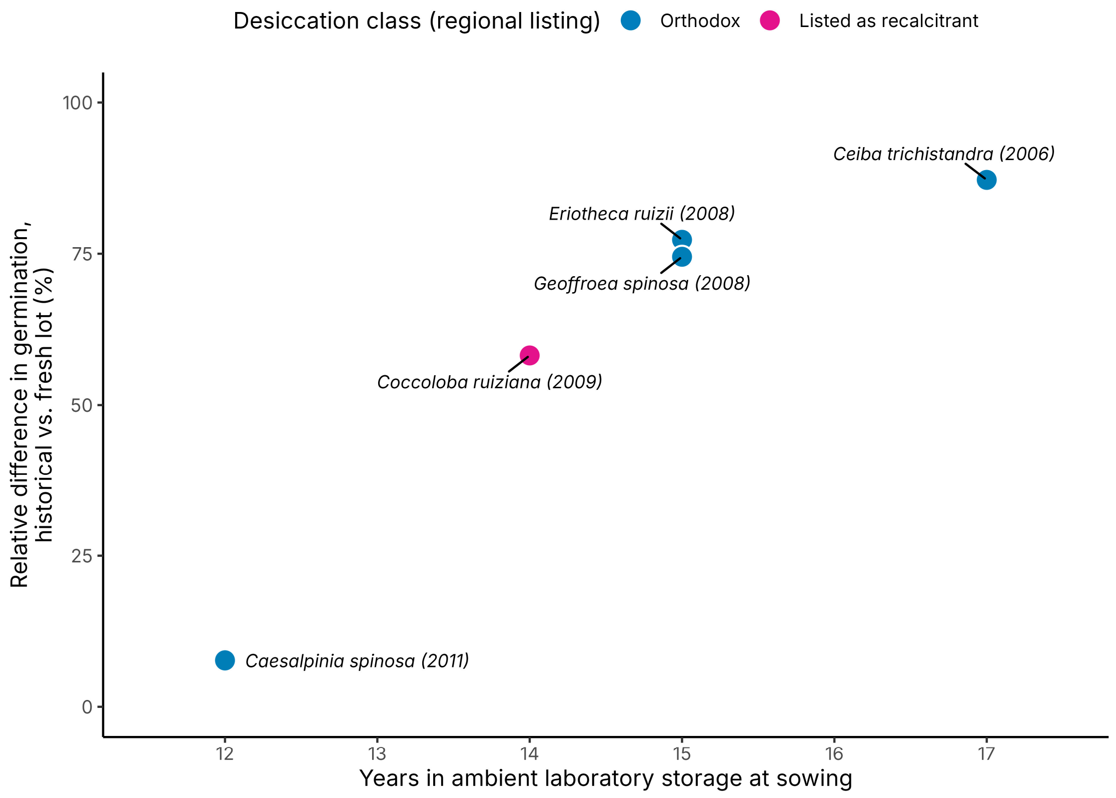

Figure S3. Exploratory comparison of the relative difference in germination between the historical and the fresh lot (as a percentage of the fresh-lot value) and the number of years each historical lot had been held under ambient laboratory conditions at sowing (collection year in parentheses). Colours show the desiccation class listed in a regional compilation [23]; *C. ruiziana*, listed there as recalcitrant, is treated as intermediate in this study. Each point represents, at the same time, a different species, collection year, storage duration and set of mother trees, and the two sowing groups are represented unequally; n = 5 species are not independent replicates. No model is fitted: the figure is not a seed-survival curve and provides no estimate of storage longevity or ageing kinetics (Spearman $\rho = 0.975$, reported descriptively).
